# MicroRNA-375-3p is implicated in carotid artery stenosis by promoting the cell proliferation and migration of vascular smooth muscle cells

**DOI:** 10.1186/s12872-021-02326-6

**Published:** 2021-10-26

**Authors:** Yuxia Yin, Zhen Cheng, Xiaoling Fu, Shishun Ji

**Affiliations:** Department of Neurosurgery, Yidu Central Hospital of Weifang, No.4138, South Linglongshan Road, Weifang, 262500 Shandong China

**Keywords:** Asymptomatic carotid artery stenosis, miR-375-3p, Diagnosis, Vascular smooth muscle cells

## Abstract

**Background:**

Atherosclerosis is the main cause of carotid artery stenosis (CAS) which mostly occurs in the elderly. In this paper, the expression level of miR-375-3p in asymptomatic CAS patients and its diagnostic value for asymptomatic CAS were investigated, and the effects of miR-375-3p on the cell proliferation and migration of vascular smooth muscle cells (VSMCs) was further explored.

**Methods:**

98 healthy subjects and 101 asymptomatic CAS patients were participated in this study. qRT-PCR was used to measure the expression level of serum miR-375-3p, and the ROC curve was established to evaluate the predictive value of miR-375-3p for asymptomatic CAS. After transfection with miR-375-3p mimic or inhibitor in vitro, cell proliferation and migration were detected by CCK-8 assay, colony formation assay, and Transwell assay, respectively. The levels of TNF-α, IL-1β, IL-6 were detected by ELISA. Western blot was used to detect the protein expression of XIAP. Finally, luciferase reporter gene assay was applied to assess the interaction of miR-375-3p with target genes.

**Results:**

The expression level of serum miR-375-3p in asymptomatic CAS patients was significantly higher than that in healthy controls, and the AUC value of ROC curve was 0.888. The sensitivity and specificity were 80.2 and 86.7%, respectively, indicating that miR-375-3p had high diagnostic value for asymptomatic CAS. In vitro cell experiments showed that up-regulation of miR-375-3p significantly promoted the proliferation and migration of VSMCs, and also promoted the generation of inflammatory factors and phenotypic transformation of VSMCs. Luciferase reporter gene assay confirmed that XIAP was a target gene of miR-375-3p and was negatively regulated by miR-375-3p.

**Conclusions:**

In this study, miR-375-3p may have a clinical diagnostic value for asymptomatic CAS patients which need further validation. Increased miR-375-3p levels in CAS may be associated with increased proliferation and migration of VSMCs via downregulation of the apoptosis inducing gene XIAP.

**Supplementary Information:**

The online version contains supplementary material available at 10.1186/s12872-021-02326-6.

## Background

According to statistics, the incidence of cerebral ischemia events (CIEs) has been increasing year by year in recent years [1]. Carotid artery stenosis (CAS) is one of the vital risk factors for CIEs, accounting for about 20% of all CIEs cases [2, 3]. CAS can be divided into asymptomatic CAS and symptomatic CAS. Asymptomatic CAS is considered to have no previous symptoms to be determined, and symptomatic CAS is determined to be associated with symptoms occurring in the preceding 6 months, while asymptomatic CAS could change from simple intimal thickening to symptomatic stenosis [4, 5]. Atherosclerosis is the main cause of CAS, with other causes including arteritis, arterial dissection, and cervical radiotherapy [6]. Vascular smooth muscle cells (VSMCs) are a crucial component of the vascular system. Under normal circumstances, VSMCs are in a homeostatic, non-proliferative state, which is mainly responsible for maintaining vascular tension and blood pressure [7, 8]. In the case of damage, VSMCs interacted with a variety of cytokines released by inflammatory cells and endothelial cells [9], leading to the massive proliferation and migration of VSMCs, which is one of the multiple processes for the progression of atherosclerotic diseases. Once asymptomatic CAS develops into symptomatic CAS, the incidence of cerebral infarction and myocardial infarction will be greatly increased. Therefore, early definite diagnosis of such lesions can provide reliable data for clinical indication of potential cerebral infarction patients and prevention of cerebral infarction.

At present, it is impossible to judge when asymptomatic CAS will appear symptoms and whether cerebral ischemia will occur. Relatively speaking, appropriate biomarkers can facilitate timely diagnosis of CAS and early intervention. MicroRNAs (miRNAs) are a class of short non-coding RNAs that affect the expression of target genes by binding to specific complementary sequences [10]. A great number of studies have declared the abnormal expression and crucial role of miRNA in human diseases [11]. Many miRNA expressions are abnormal in atherosclerosis, including miR-181b, miR-92a, miR-126, etc., indicating that these miRNAs may be involved in the progression of the disease [12]. Chen et al. reported that high-level of miR-92a has the ability to distinguish asymptomatic CAS patients from healthy control people, showing its value as a diagnostic marker of asymptomatic CAS [13]. Zhang et al. reported that miR-106b-5p was abnormally expressed in asymptomatic CAS patients, and high expression of miR-106b-5p was significantly correlated with the occurrence of CIEs in patients [14]. miR-375-3p was originally found to be abnormally expressed in a variety of cancers. Cen et al. reported that miR-375-3p acts as a tumor suppressor gene in head and neck squamous cell carcinoma (HNSCC) by regulating the tumor-associated gene FN1, VEGFA [15]. In a recent study of Qiu et al., it was claimed that overexpression of miR-375 promoted the development of atherosclerosis by activating inflammatory response and foam cell formation [16]. However, the effects of miR-375-3p on CAS and VSMCs remained to be further studied. X-linked inhibitor of apoptosis protein (XIAP) is a kind of apoptosis inhibitor, which has become the most effective caspase inhibitor by directly binding and inhibiting caspase target [17]. It is reported that XIAP exists in endothelial cells and has anti-atherosclerosis effect [18, 19]. It will be interesting to study whether XIAP is associated with miR-375-3p in CAS for exploring the pathological mechanism of CAS. In the present study, we detected the level of serum miR-375-3p in asymptomatic CAS patients, evaluated the diagnostic value of miR-375-3p in CAS, and further explored the effects of miR-375-3p on the proliferation and migration of VSMCs and the possible mechanisms.

## Methods

### Subject recruitment and data collection

This study was performed in accordance with the ethical standards as laid down in the Declaration of 1964 Helsinki and its later amendments. And the study has been approved by the Ethics Committee of Yidu Central Hospital of Weifang. All subjects have signed the informed consent. A total of 101 asymptomatic CAS patients took part in this study. Inclusion criteria were based on previously published literature [20]: (1) the degree of ipsilateral internal carotid artery stenosis was greater than 50%, (2) asymptomatic status was determined by history review of patients, physical examination, and investigation of the National Institutes of Health Stroke Scale (NIH-SS). Exclusion criteria were patients with a history of stroke, congestive heart failure, inflammatory disease, transient ischemic attack, and malignant tumors [13]. In addition, 98 subjects who came to physical examination center of the hospital for health examination and were excluded a history of cardiovascular disease, and malignancy were used as healthy controls. All subjects underwent color Doppler ultrasound and angiography to determine the degree of stenosis, and only patients with stenosis less than 20% were included in the healthy control group [21]. Details of the subjects inclusion procedure in this study are provided in Additional file 1. Venous blood of all subjects was collected, and supernatant was taken after centrifugation and stored at − 80 °C for later use. The basic data and clinical characteristics of participants were recorded for subsequent analysis.

### Cell culture and transfection

Human vascular smooth muscle cells (VSMCs) were acquired from SIBCB and cultured in DMEM supplemented with 10% FBS (Hyclone, USA) and 1% Penicillin/streptomycin. The expression level of miR-375-3p in VSMCs was regulated by in vitro transfection technique. Briefly, VSMCs from passage 4 to 8 were seeded into 12-well plate and incubated overnight. According to the manufacturer’s protocols, VSMCs were transfected with miR-375-3p mimic and miR-375-3p inhibitor which were synthesized by GenePharma (Shanghai, China) using Lipofectamine 3000 (Invitrogen, USA) for 48 h.

### RNA extraction and qRT-PCR

Total RNAs were extracted by Trizol according to the product instruction. PrimeScript™ RT reagent Kit and SuperScript II Reverse Transcriptase kit were used for reverse transcription. In the Applied Biosystems 7900 Real-Time PCR System, the expression level of miRNAs or mRNAs were detected using the miScript SYBR Green PCR kit. With U6 and GAPDH as internal parameters, the relative expressions of RNAs were calculated by 2^−ΔΔCt^ method.

### Cell viability assay

The cell proliferation of VSMCs was assessed by cell counting kit-8 (CCK-8) assay. The transfected VSMCs in logarithmic growth phase were seeded into 96-well plates at a density of 5 × 10^4^ cells/well, and the proliferation ability of VSMCs was detected at 0 h, 24 h, 48 and 72 h. At every time point, 10 µL of CCK-8 working solution was added to the plates and cultured in the dark for 1 h. Subsequently, the OD value at 450 nm was determined using a microplate analyzer (SpectraMax; Molecular Devices, LCC). Each time point was performed in triplicate.

### Colony formation assay

VSMCs treated with miR-375-3p mimic, miR-375-3p inhibitor or miR-NC were evenly seeded into 6-well plate at a density of 5 × 10^2^ cell/well, and cells were cultured in conventional culture conditions. After 2 weeks of incubation, the culture media was discarded, and the cells were washed three times with PBS. Cells were fixed with 4% paraformaldehyde for 30 min, and then stained with 0.5% crystal violet for 20 min. Finally, the number of colonies were counted with inverted microscope. Each colony is defined as more than 50 cells.

### Cell migration assay

Transwell assay was performed for cell migration evaluation. In brief, the transfected VSMCs were collected and dispersed in FBS-free medium and then inoculated into the upper Transwell chamber. At the same time, medium containing FBS was added into the lower Transwell chamber. After the above chambers were placed in an incubator for 24 h, cells migrated to a flat plane below were fixed and stained, and five fields were randomly selected for cell counting under an inverted fluorescence microscope (Olympus Corporation, Japan).

### Enzyme-linked immunosorbent assay (ELISA)

VSMCs in logarithmic growth phase were inoculated into 6-well plate at a density of 5 × 10^4^ cells/well. After 48 h of cell culture, the supernatants were collected. The concentrations of TNF-α, IL-1β, and IL-6 in cell supernatants were measured by ELISA using ELISA kits (Invitrogen, Carlsbad, CA, USA) in accordance with manufacturer’s protocols.

### Western blot

The total protein of VSMCs was extracted by ice-cold RIPA buffer (Beyotime, Shanghai, China), and the protein concentration was quantified by BCA assay kit (Thermo Fisher Scientific, Rockford, IL, USA). After adjusting the protein concentration, sodium dodecyl sulfate polyacrylamide gel electrophoresis (SDS-PAGE) was performed, and the protein samples were transferred to polyvinylidene fluoride membranes. The membranes were blocked with 5% non-fat dry milk in TBS with 0.1% of Tween 20 for 2 h. Subsequently, the membranes were incubated with primary antibodies against XIAP at 4 °C overnight. Next, the membranes were incubated with secondary antibodies at room temperature for 45 min. Finally, protein signals were visualized by electrochemiluminescence detection system.

### Luciferase reporter gene assay

Target gene prediction was performed using the Targe-Scan 7.0 online program, and it was found that miR-375-3p had complementary binding sites with the 3’-UTR regions of XIAP, and this was verified by luciferase reporter gene assay. The 3’-UTR sequence fragments of XIAP were cloned into the pGL3 luciferase reporter gene vector, and the wild-type reporter vector XIAP-3’-UTR-WT and mutant type reporter vector XIAP-3’-UTR-MUT were constructed. VSMCs were seeded into 24-well plates at a density of 1 × 10^5^ cells per well and cultured overnight until the cell density per well reached more than 60 %. Subsequently, the above vectors and miR-NC miR-375-3p mimic or miR-375-3p inhibitor were co-transfected into the cells using Lipofectamine 3000 (Invitrogen, USA) according to the product instructions. After 48 h of transfection, cells were collected and luciferase activity in each group was measured using a dual luciferase reporting system (Promega, USA). Renal luciferase intensity was used as the internal reference.

### Statistical analysis

Data were analyzed by SPSS. The normality of experimental data was analyzed by Kolmogorov–Smirnov (K–S) normality test. The differences between groups were detected by Student t test and one-way ANOVA. ROC curve was drawn to assess the diagnostic value of miR‑375‑3p in asymptomatic CAS. *P* < 0.05 was considered statistically significant. The data conforming to the normal distribution were expressed as mean ± standard deviation (SD). Each experiment was performed in triplicate.

## Results

### Clinical characteristics of samples

The general information and clinical data of the subjects are shown in Table 1. There was no significant difference in gender, age, BMI, FBG and other indicators in healthy controls and CAS patients (*P* > 0.05). Compared with the healthy population, the degree of carotid artery stenosis in asymptomatic CAS patients was 67.59 ± 11.15.

**Table 1:** Clinical data of the study population

Characteristics	Healthy individuals (n = 98)	CAS patients (n = 101)	*P* value
Gender (male and female)	47/51	47/54	0.607
Age (years)	64.93 ± 7.65	63.15 ± 8.25	0.116
BMI (kg/m^2^)	22.89 ± 2.80	22.90 ± 3.00	0.966
FBG (mg/dL)	92.14 ± 17.50	95.16 ± 18.09	0.234
TC (mg/dL)	191.43 ± 4.77	191.41 ± 3.84	0.979
TG (mg/dL)	122.02 ± 12.94	119.98 ± 13.05	0.271
HDL (mg/dL)	48.90 ± 3.77	48.80 ± 4.18	0.873
LDL (mg/dL)	111.93 ± 6.33	112.67 ± 7.01	0.437
SBP (mm Hg)	127.93 ± 11.80	130.64 ± 13.78	0.138
DBP (mm Hg)	77.67 ± 9.17	80.30 ± 10.43	0.061
Degree of carotid artery stenosis	14.07 ± 3.71	67.59 ± 11.15	< 0.001

Data are expressed as n or mean ± standard deviation

CAS, carotid artery stenosis; BMI, body mass index; FBG, fasting blood glucose; TC, total cholesterol; TG, triglycerides; HDL, high-density lipoprotein; LDL, low density lipoprotein; SBP, systolic blood pressure; DBP, diastolic blood pressure

### Serum level of miR-375-3p

**Fig. 1:**
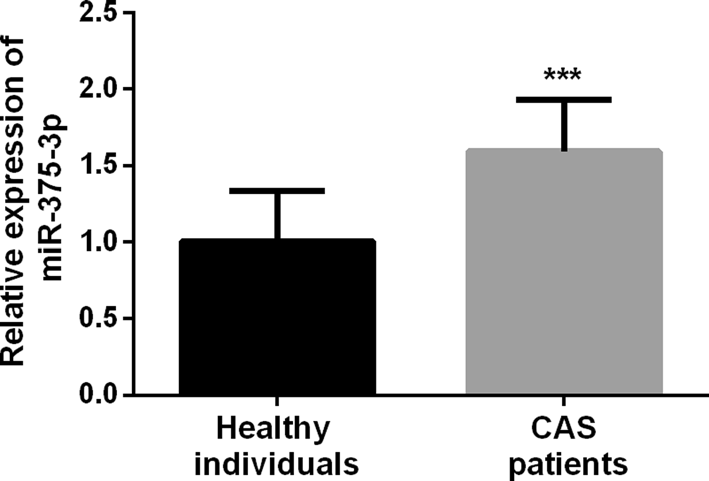
Relative expression of serum miR-375-3p in 101 asymptomatic CAS patients and 98 healthy controls (^***^P < 0.001)

Serum levels of miR-375-3p were significantly increased in asymptomatic CAS patients compared with healthy controls (Fig. 1, *P* < 0.001), indicating that the abnormal expression of miR-375-3p may be related to the occurrence of CAS.

### Diagnostic value of miR‑375‑3p in asymptomatic CAS

**Fig. 2:**
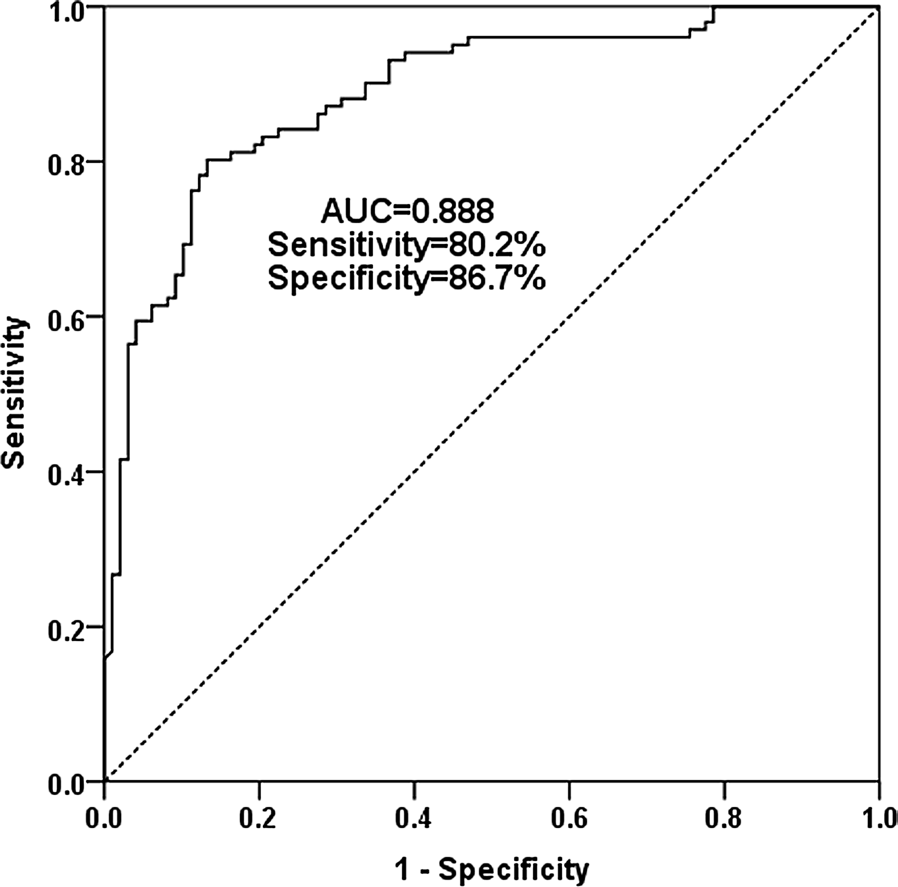
ROC curve analysis was performed to estimate the diagnostic value of miR-375-3p for CAS

As shown in Fig. 2, the curve had an AUC value of 0.888 with the sensitivity and specificity of 80.2% and 86.7% at the cut-off value of 1.31, revealing that miR-375-3p had the ability to distinguish asymptomatic CAS patients from healthy controls, suggesting that miR-375-3p may have certain diagnostic value for asymptomatic CAS.

### Effects of miR-375-3p on proliferation and migration of VSMCs functions

**Fig. 3:**
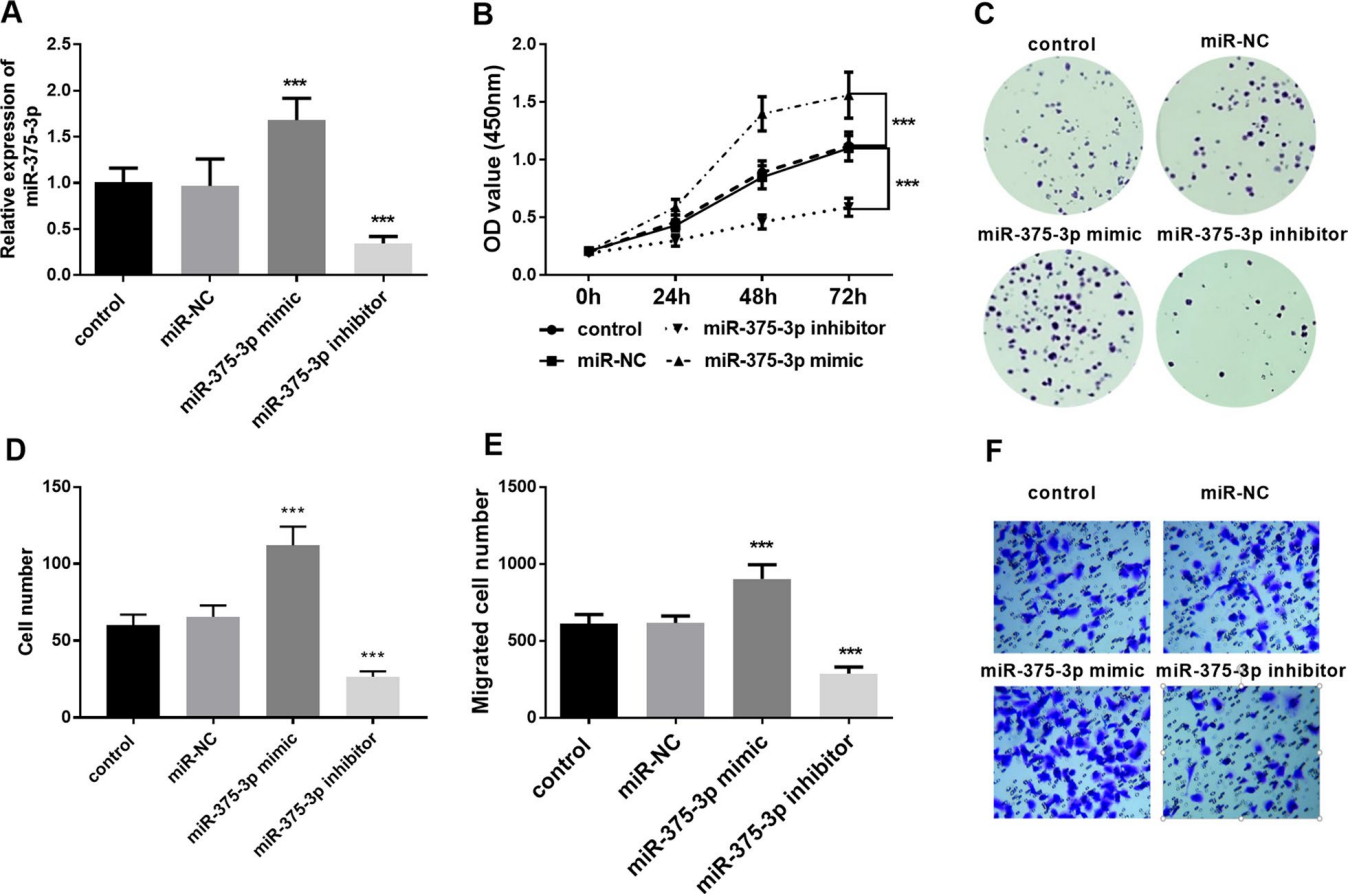
Effects of miR-375-3p on cell functions. **A** Expression level of miR-375-3p in VSMCs was regulated by cell transfection (^***^P < 0.001). **B** CCK-8 assay and **C** Colony formation assay showed that up-regulation of miR-375-3p promoted the cell proliferation (^***^P < 0.001). **D** Cell number of colony formation assay (^***^P < 0.001). **E**, **F** Cell migration assay revealed that up-regulation of miR-375-3p promoted the cell migration (^***^P < 0.001). Each experiment was performed in triplicate

Expression of miR-375-3p was significantly upregulated by transfected with miR-375-3p mimic, while the level of miR-375-3p was decreased after transfection with miR-375-3p inhibitor (Fig. 3A, P < 0.001). CCK-8 assay and colony formation assay both confirmed that compared with the control group, up-regulation of miR-375-3p significantly promoted the proliferation of VSMCs, while down-regulation of miR-375-3p had the opposite result (Fig. 3B–D, *P* < 0.001). Moreover, Transwell assay also verified that overexpression of miR-375-3p significantly increased the number of migratory cells, while down-regulation of miR-375-3p decreased the number of migratory cells (Fig. 3E, F, *P* < 0.001). Based on above cell experiment results, we showed that the up-regulation of miR-375-3p expression may be associated with the development of CAS by promoting the proliferation and migration of VSMCs.

### Effects of miR-375-3p on inflammation and contractility of VSMCs

**Fig. 4:**
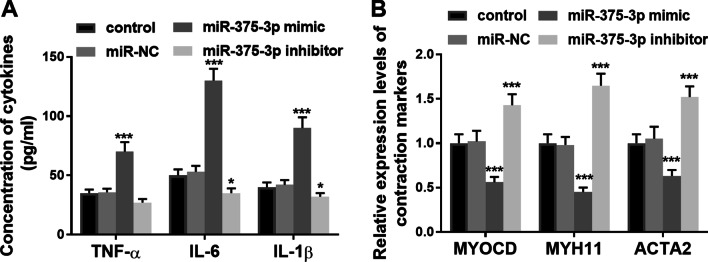
Effects of miR-375-3p on inflammation and contractility of VSMCs. **A** Overexpression of miR-375-3p promoted the levels of inflammatory factors (^**^P < 0.01). **B** Overexpression of miR-375-3p down-regulated the level of contraction markers of VSMCs (^*^P < 0.05). Each experiment was performed in triplicate

In the supernatants of VSMCs, it was observed that up-regulation of miR-375-3p promoted the generation of TNF-α, IL-1β and IL-6, while inhibition of miR-375-3p significantly declined the levels of inflammatory factors, indicating that overexpression of miR-375-3p may promote the inflammatory response of VSMCs (Fig. 4A, *P* < 0.05). Besides, it was found that overexpression of miR-375-3p decreased the mRNA expression of Myocardin (MYCOD), myosin heavy chain 11 (MYH11) and actinalpha2 (ACTA2), while the silence of miR-375-3p could reverse the above phenomenon (Fig. 4B, *P* < 0.001).

### XIAP was the target gene of miR-375-3p in VSMCs

**Fig. 5:**
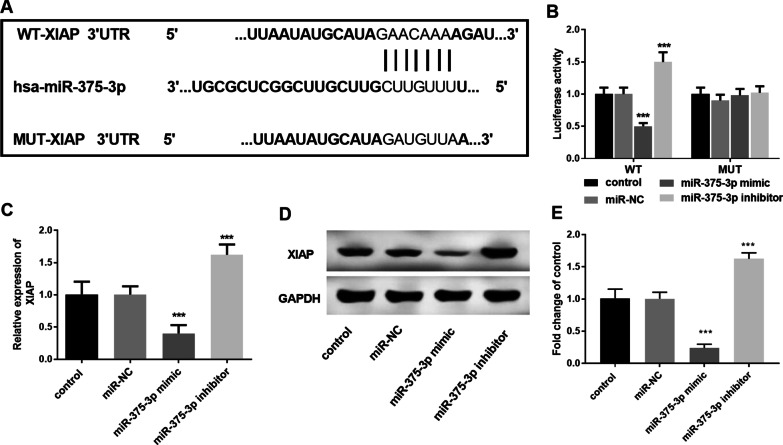
XIAP was the target gene of miR-375-3p. **A** Complementary sequences of miR-375-3p and XIAP. **B** Luciferase reporter gene assay was used to evaluate the relationship of miR-375-3p and XIAP (^***^P < 0.001). **C** XIAP level was measured by qRT-PCR (^***^P < 0.001). **D**, **E** Western blot analysis of XIAP. GAPDH was analyzed as an internal control. Bands were quantified by densitometry and normalized to the control values. Each experiment was performed in triplicate

The complementary sequence of miR-375-3p and XIAP is shown in Fig. 5A. The luciferase reporter gene results showed that the luciferase activity of XIAP-3’-UTR-WT was significantly decreased after transfection with miR-375-3p mimic (Fig. 5B, *P* < 0.001). In addition, neither miR-375-3p mimic nor inhibitor transfection had any effect on XIAP-3’-UTR-MUT. Similarly, in VSMCs, qRT-PCR showed that the expression level of XIAP was significantly decreased after upregulation of miR-375-3p, while the expression of XIAP was significantly upregulated after transfection with miR-375-3p inhibitor (Fig. 5C, *P* < 0.001). Western blot assays revealed that miR-375-3p inhibitor decreased the protein levels of XIAP, while miR-375-3p mimic reversed these effects (Fig. 5D, E, *P* < 0.001). Original cropped Western blot gel image for XIAP and GAPDH was shown in Additional file 2.

## Discussion

MiRNAs are widely involved in many biological processes in cells and are related to the mechanism of cardiovascular diseases [22]. Besides, miRNAs are relatively short in length and high in abundance, and could be easily obtained from saliva, urine, blood, and other body fluids [23, 24]. As biomarkers, miRNAs are an ideal means to detect the occurrence of cardiovascular diseases. For CAS, the carotid artery is the most important blood vessel connecting various tissues and organs of the head with the heart, and its importance is self-evident [25]. Under the current conditions, the clinical diagnosis of CAS is mainly based on imaging methods, including carotid ultrasound, NMR angiography and CT angiography [26, 27]. And angiography would cause certain damage to the body to a certain extent, such as requiring surgery, radiation exposure, allergic reaction induced by contrast agents [28]. Therefore, it is urgent and vital to find sensitive and specific biomarkers for the diagnosis of disease.

In this study, serum level of miR-375-3p in asymptomatic CAS patients was significantly increased in comparison with healthy controls, which confirmed that miR-375-3p played a crucial role in CAS. Zhang et al. reported that serum miR-375 in patients with hypertension was increased [29]. A previous report on Tanshinone IIA by Chen et al. showed that the level of miR-375 was elevated in the aorta of a mouse model of atherosclerosis, and TNA activated KLF4 by inhibiting miR-375, thereby enhancing macrophage autophagy and alleviating atherosclerosis [30]. The results of these studies are consistent with the results of the present study. Meanwhile, the ROC curve verified the potential diagnostic value of miR-375-3p in asymptomatic CAS. The higher AUC value, sensitivity, and specificity of miR-375-3p indicated that miR-375-3p had the ability to distinguish asymptomatic CAS patients from healthy people. Therefore, we believe that early and correct diagnosis of disease is not only conducive to the timely detection, but also conducive to disease treatment. Early diagnosis of CAS, especially for asymptomatic CAS patients with no typical clinical features, is of great significance to prevent the cardiovascular and cerebrovascular diseases.

The direct cause of most CAS is atherosclerosis, while the abnormal biological functions of VSMCs have been shown to be involved in atherosclerosis. It was reported that the main component of atherosclerotic plaques is VSMCs, and the proliferation of VSMCs is a key step in plaque formation. Zhang et al. showed that miR-148b was down-regulated in atherosclerotic plaques, and exogenous miR-148b mimics antagonized the proliferation of VSMCs by targeting HSP90 [31]. Han et al. reported that the expression of miR-145 was decreased in CAS patients, while the up-regulation of miR-145 significantly inhibited the proliferation of VSMCs in vitro [32]. In this study, VSMCs were transfected with exogenous miR-375-3p mimics or inhibitors to explore the influence of miR-375-3p on VSMCs. The results revealed that the up-regulation of miR-375-3p expression significantly promoted the proliferation and migration of VSMCs, while down-regulation of miR-375-3p could significantly inhibit the proliferation and migration of VSMCs. This result indirectly explains the function of miR-375-3p in the development of CAS from the cellular level, and at the same time, it also provides us with a relevant direction to solve CAS. Subsequently, we also found that upregulation of miR-375-3p promoted the production of inflammatory factors. Moreover, highly expressed miR-375-3p significantly reduced the mRNA levels of MYOCD and downstream contracted proteins. Phenotypic transformation of VSMCs plays an important role in cardiovascular disease [33]. The transformation of VSMCs from contractile type to synthetic type is often accompanied by the enhancement of cell proliferation and migration ability and the increase of pro-inflammatory cytokines secretion, which leads to a series of vascular diseases [34, 35]. In this study, the decrease of in vitro levels of MYOCD, MYH11 and ACTA2 suggested the decrease of VSMCs contractility. This result was consistent with the results of cell proliferation and migration. In fact, many related studies have been carried out to control the progression of CAS by aiming to control the biological function of VSMCs. For example, Hu et al. found that miR-125a-3p mimics effectively inhibited the proliferation and migration of VSMCs, while in a rat model with carotid artery injury, overexpression of miR-125a-3p reduced the formation of new intima, thus effectively inhibiting vascular stenosis [36]. Combined with previous studies, we think that controlling the cellular function of VSMCs may become an important method for the treatment of CAS.

In addition, we preliminarily confirmed that XIAP was the target gene of miR-375-3p according to targe-scan data analysis, and the subsequent luciferase reporter gene assay also confirmed this result. Meanwhile, in VSMCs, the expression of XIAP in cells was significantly decreased after transfection of the miR-375-3p mimics. The above results confirmed that XIAP was a target gene of miR-375-3p and was negatively regulated by miR-375-3p. In a study on toxic epidermal necrolysis (TEN) conducted by Zhang et al., they found that miR-375-3p in exosomes induced keratinocytes apoptosis by targeting XIAP, thereby promoting the development of TEN [37]. XIAP is the only endogenous caspase inhibitor in mammals, which can inhibit the execution of apoptosis [38]. A previous study showed that overexpression of miR-122 in human aortic endothelial cells directly targeting and inhibiting XIAP produces pro-apoptotic effects that contribute to conditions that promote atherosclerosis development [39]. According to the above studies, we preliminarily confirmed that in VSMCs, miR-375-3p promoted cell proliferation and migration by targeting XIAP, thus promoting the development of CAS.

There are some limitations to this study. Firstly, due to the small sample size and single source of cases, potential deviations may occur in the process. To solve this problem, we need to expand the sample size in future experiments. Secondly, after the asymptomatic CAS patients were included, we did not examine the coronary arteries or peripheral arteries of these patients and could not rule out whether these arteries had atherosclerotic lesions. Therefore, it is impossible to rule out whether the high level of miR-375-3p in asymptomatic CAS patients is influenced by hidden lesions. These should have been taken into account in the initial design of the experiment.

## **Conclusions**

In conclusion, it was confirmed that the high expression of miR-375-3p in the serum of asymptomatic CAS patients had certain diagnostic value for CAS. Overexpression of miR-375-3p may promote the proliferation, migration, inflammatory response and phenotypic transformation of VSMCs by targeting XIAP. The results of this study provide a certain experimental basis for understanding the mechanism of miR-375-3p on CAS.

## Supplementary Information

**Additional file 1**. Flowchart of patient recruitment.

**Additional file 2**. Original cropped Western blot gel image for XIAP and GAPDH.

## Data Availability

All data generated or analyzed during this study are included in this article and its supplementary material files. Further enquiries can be directed to the corresponding author (Shishun Ji, E-mail: ashun09428@163.com).

## Abbreviations

CAS: Carotid artery stenosis
CIEs: Cerebral ischemia events
VSMCs: Vascular smooth muscle cells
miRNAs: MicroRNAs
NIH-SS: National Institutes of Health Stroke Scale
CCK-8: Cell counting kit-8
XIAP: X-linked inhibitor of apoptosis protein
SD: Standard deviation
