# Supplementary figures and images for: MicroRNA-375-3p is implicated in carotid artery stenosis by promoting the cell proliferation and migration of vascular smooth muscle cells
Source: BMC Cardiovasc Disord. 2021 Oct 26;21:518. doi: 10.1186/s12872-021-02326-6 (PMC8549333; doi:10.1186/s12872-021-02326-6)

**Supplementary Materials: Flowchart of Patient Recruitment**
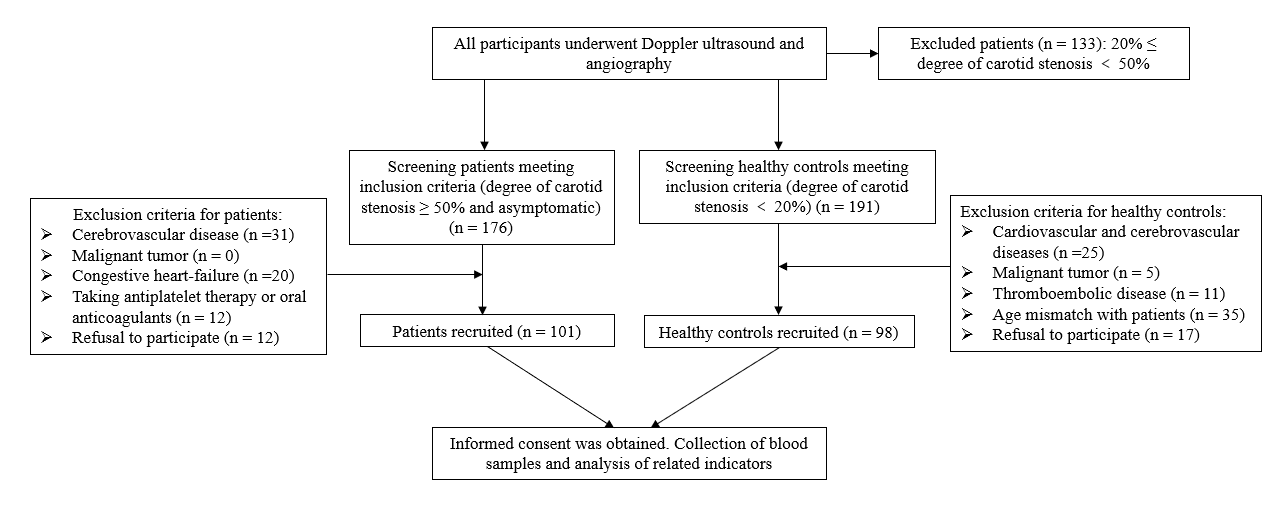

Supplement: Supplementary file 1 — Additional file 1. Flowchart of patient recruitment. [file 12872_2021_2326_MOESM1_ESM.docx]
