# Supplementary material for: MicroRNA-375-3p is implicated in carotid artery stenosis by promoting the cell proliferation and migration of vascular smooth muscle cells
Source: BMC Cardiovasc Disord. 2021 Oct 26;21:518. doi: 10.1186/s12872-021-02326-6 (PMC8549333; doi:10.1186/s12872-021-02326-6)
Supplement: Supplementary file 2 — Additional file 2. Original cropped Western blot gel image for XIAP and GAPDH. [file 12872_2021_2326_MOESM2_ESM.docx]

Original cropped Western blot gel for XIAP and GAPDH. Molecular masses of standard proteins are listed to the left of the gel.


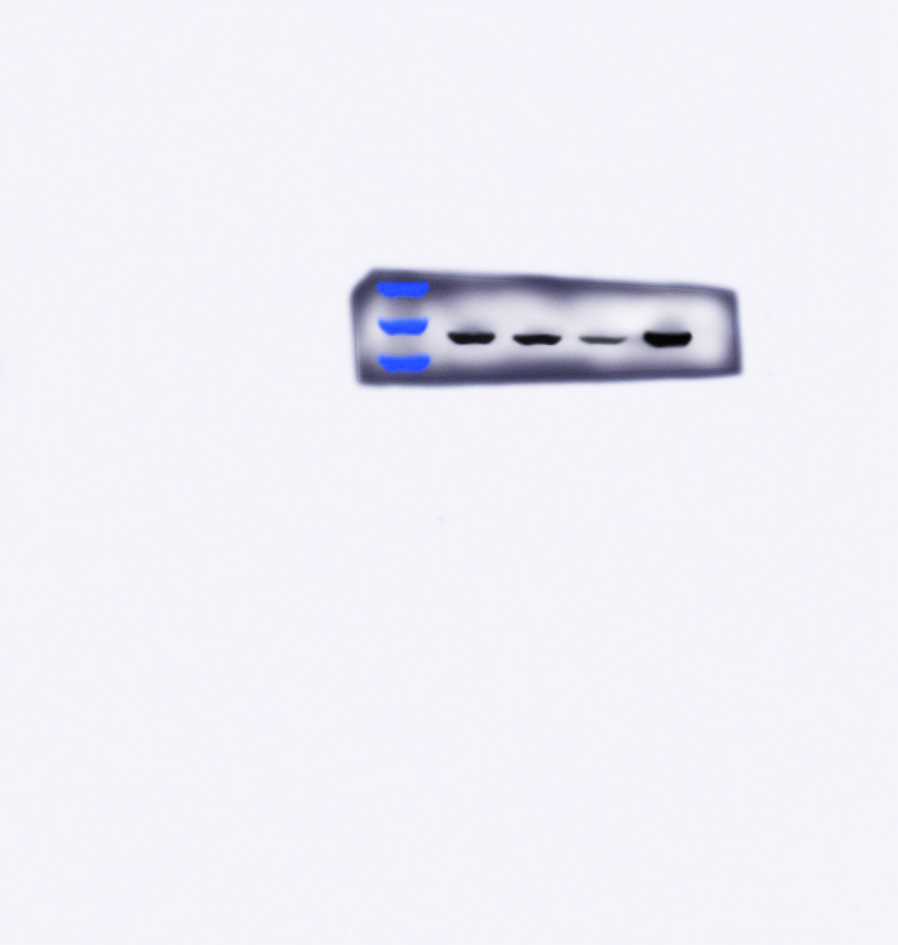


KDa

70

60

50

XIAP


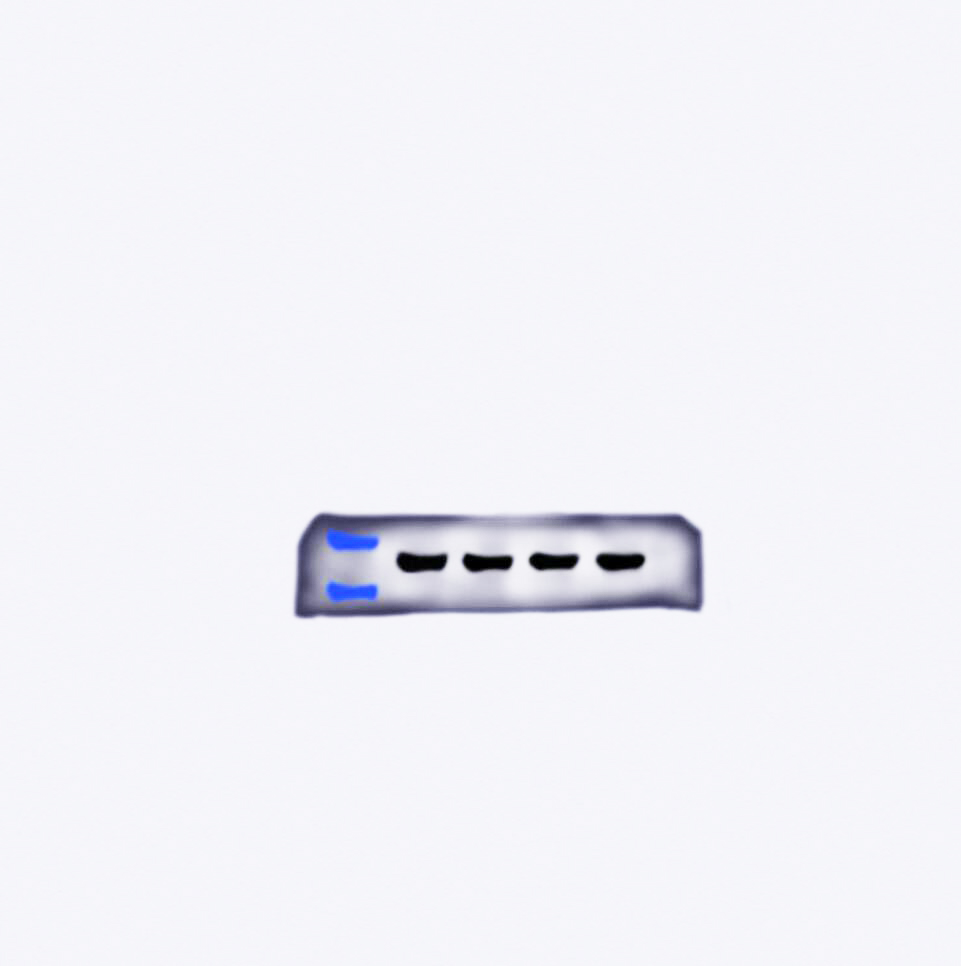


KDa

40

30

GAPDH
